# SiteFind: A software tool for introducing a restriction site as a marker for successful site-directed mutagenesis

**DOI:** 10.1186/1471-2199-6-22

**Published:** 2005-12-01

**Authors:** Paul M Evans, Chunming Liu

**Affiliations:** 1 Department of Biochemistry and Molecular Biology, University of Texas Medical Branch, Galveston, USA; 2 Sealy Center for Cancer Cell Biology, University of Texas Medical Branch, Galveston, USA

## Abstract

**Background:**

Site-directed mutagenesis is a widely-used technique for introducing mutations into a particular DNA sequence, often with the goal of creating a point mutation in the corresponding amino acid sequence but otherwise leaving the overall sequence undisturbed. However, this method provides no means for verifying its success other than sequencing the putative mutant construct: This can quickly become an expensive method for screening for successful mutations. An alternative to sequencing is to simultaneously introduce a restriction site near the point mutation in manner such that the restriction site has no effect on the translated amino acid sequence. Thus, the novel restriction site can be used as a marker for successful mutation which can be quickly and easily assessed. However, finding a restriction site that does not disturb the corresponding amino acid sequence is a time-consuming task even for experienced researchers. A fast and easy to use computer program is needed for this task.

**Results:**

We wrote a computer program, called SiteFind, to help us design a restriction site within the mutation primers without changing the peptide sequence. Because of the redundancy of genetic code, a given peptide can be encoded by many different DNA sequences. Since the list of possible restriction sites for a given DNA sequence is not always obvious, SiteFind automates this task. The number of possible sequences a computer program must search through increases exponentially as the sequence length increases. SiteFind uses a novel "moving window" algorithm to reduce the number of possible sequences to be searched to a manageable level. The user enters a nucleotide sequence, specifies what amino acid residues should be changed in the mutation, and SiteFind generates a list of possible restriction sites and what nucleotides must be changed to introduce that site. As a demonstration of its use, we successfully generated a single point mutation and a double point mutation in the wild-type sequence for Krüppel-like factor 4, an epithelium-specific transcription factor.

**Conclusion:**

SiteFind is an intuitive, web-based program that enables the user to introduce a novel restriction site into the mutated nucleotide sequence for use as a marker of successful mutation. It is freely available from

## Background

There are several methods available for mutagenesis: 1) to isolate single strand template DNA and then create the mutation with one complementary primer [1]; 2) design two sets of PCR primers that overlap the mutation site, amplify the template by two PCR reactions and then clone the two PCR fragments and the vector by three piece ligation [2]; 3) Site-directed mutagenesis using the QuikChange method [3-5]. All of these *in vitro *mutagenesis methods require careful design of one or more primers that cover the mutation site. Currently, QuikChange site-directed mutagenesis is the method of choice. This method requires two complementary oligonucleotide primers flanking the desired mutated nucleotide on both the sense and anti-sense strands. Furthermore, each primer must contain one to several base-pair changes within the desired region. PCR is then performed using these primers along with the gene of interest, which was previously inserted into a vector containing an antibiotic resistance gene. The extension step of the polymerase chain reaction is given sufficient time to replicate the entire circular DNA construct, with the reaction eventually ending where it started. After several rounds of PCR, the resulting mixture of newly-synthesized mutant constructs and template DNA is incubated with a methylation-specific endonuclease to remove the wild-type template DNA which contains methylated nucleotides. The mixture is then transformed into competent bacteria, plated on an antibiotic-containing medium, and grown overnight to in order to allow individual colonies to grow.

However, since the bacteria was transformed with a complex mixture of undigested template DNA, successful point mutant copies of the template, and PCR side-products, it becomes difficult to determine which colonies contain the desired mutant construct. Restriction enzyme digestion of plasmid DNA extracted from each colony can differentiate between correct and aberrant PCR products, but it cannot distinguish between bacteria transformed with template DNA and bacteria transformed the with desired point mutant. Instead, plasmid DNA extracted from each colony must be sent to a sequencing laboratory and the sequence manually scanned for a successful mutation. If the number of colonies containing template DNA is high relative to the total number of colonies, this can be an expensive and time-consuming process.

A simple method to confirm the presence of a point mutation prior to sequencing is to design the mutation of the sequence such that it introduces a novel restriction site, taking advantage of the redundancy of the genetic code [6-8]. Thus plasmid DNA extracted from each colony can be digested with the appropriate restriction enzyme and then run on a DNA gel to check for the presence of a band not found in the template DNA. However, finding the correct set of mutations to the DNA sequence in order to introduce a restriction site without disturbing its corresponding amino acid sequence is not always a trivial task, requiring the investigator to manually generate hundreds of possible DNA sequences and then scan them for restriction sites. Even for an experienced molecular biologist, it will take time and luck to find a suitable site. SILMUT, a program written and published several years ago, can be used to discover such diagnostic restriction sites [9]. The user enters a short amino acid sequence, and SILMUT determines if any of 30 of the most common, 6 bp restriction sites can introduced within that sequence. To make this task much faster and less error-prone, we wrote our own, web-based computer program, called SiteFind.

In some cases, however, silent mutations in the coding sequence can have a drastic effect on the translation rate. Thus, the user must be alert to the possibility of codon bias in the organism where this sequence will be expressed.

## Implementation

### SiteFind overview

The ultimate goal of SiteFind is to search a given nucleotide sequence for any possible restriction sites that can be introduced without disturbing the amino acid sequence that it codes for. For example, the sequence CTCGAA codes for the amino acid sequence LE, or leucine-glutamate, but does not possess any common restriction site. However, by simply changing the last Adenine to a Guanine, the sequence becomes CTCGAG, which is the restriction site for *XhoI*. At the same time, the amino acid sequence is preserved, since both GAA and GAG code for glutamate. For such a short sequence, the necessary mutations to introduce a restriction site may be obvious, but SiteFind can quickly search through much longer sequences, where potential restriction sites may be hidden in long sequence of nucleotides. We found that on the average end-user personal computer, SiteFind can handle sequences of up to approximately 400 bp.

SiteFind was designed with the purpose of introducing a restriction site into a nucleotide sequence as a marker for successful point mutation via site-directed mutagenesis. Consistent with this purpose, the user can specify which amino acids should be changed in the peptide sequence and then select the potential restriction site closest to the point mutation. Ideally, these two will overlap, but this is not always possible. A novel restriction site within a few nucleotides of the point mutation is often sufficient to use as a marker.

### Algorithm optimizations

**Figure 1:**
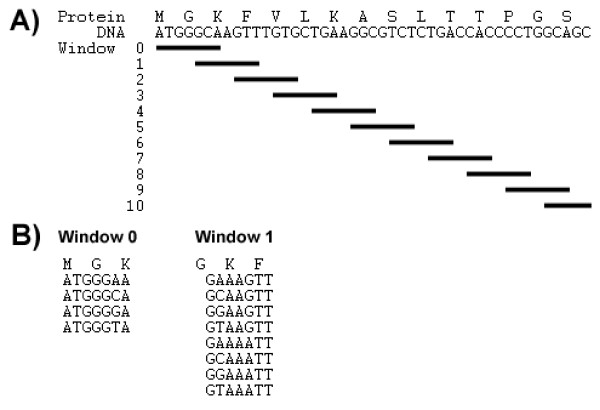
**Moving window algorithm**. a) Example of how the algorithm is implemented with a 4 nucleotide restriction site. Each window is therefore 7 nucleotides and each successive window is shifted forward 4 nucleotides, ensuring minimal overlap. b) Example of all the possible sequences generated for each of the first two search windows using the moving window algorithm.

The redundancy in the genetic code means that as the length of a given amino acid sequence increases, the number of possible DNA sequences that can code for that sequence increases exponentially. Since the amino acid serine can be represented by six different codons, this means that a sequence of four serines can be represented by 6^4 ^(1296) different DNA sequences. To substantially reduce the number sequences to scan by our program, SiteFind uses a "moving window" algorithm (See Fig. 1A). The "moving window" algorithm effectively breaks up a long nucleotide sequence into a series of short, non-redundant sequences that can be then searched individually. Thus, a long amino acid sequence with millions of possible nucleotide sequences can be converted into 10 or so "windows", each with only a few hundred possible sequences.

The size of each "window" is determined by the length of the longest restriction site the user is searching for. In general, for a given restriction site of *n *nucleotides, the window must be at least *2n-1 *nucleotides long. SiteFind then shifts the window only enough to ensure overlap between windows such that any possible restriction site is found, meaning that the window is shifted forward no more than *n *nucleotides (See Fig. 1B). This process is then repeated until the entire nucleotide sequence is traversed.

SiteFind was originally written in C++ as a simple command-line tool for in-house use. We subsequently rewrote the program as a Java applet embedded in a HTML web document, giving it a more intuitive, graphical interface and posted it on our institutional website. The source code to our Java applet is freely available and is released under the GPL [10]. SiteFind was written using TextPad v4.7.3 [11] and compiled with the Java 1.4.2 SDK [12]. The website was designed with Microsoft FrontPage.

## Results

### Using sitefind

**Figure 2:**
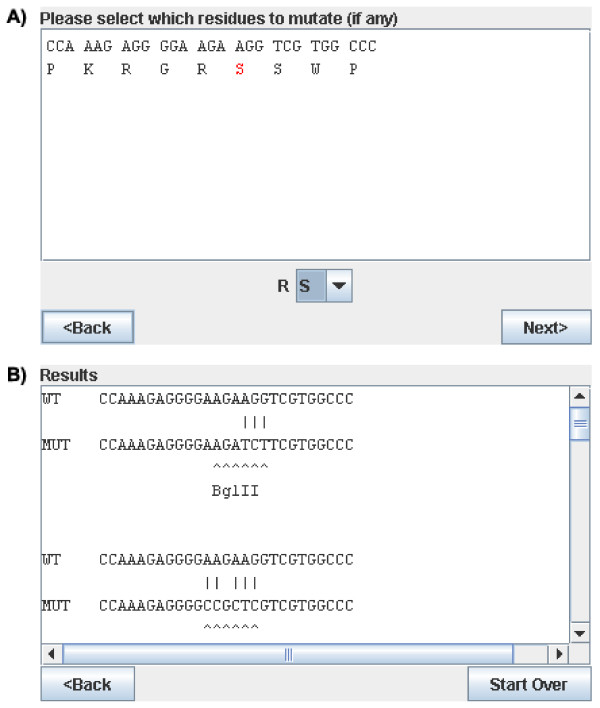
**SiteFind Screenshots**. a) Sample input, showing translated nucleotide sequence and a mutant residue highlighted in red. b) Sample output, showing a novel BglII site discovered within the sequence.

SiteFind was designed to have an intuitive interface, with each step necessary to specify the search conditions presented in a separate window. A button labeled "Next" at the bottom right hand corner of each window allows the user to progress to the next step. The SiteFind applet loads in a browser once its webpage is visited and prints out a simple message identifying the program name and creator. To begin, the user clicks "Next". The first window prompts the user to enter a short segment (preferably at least 15 nucleotides) of the wild-type DNA sequence, covering the region where a mutation is desired. The user is then prompted to select the correct reading frame for the sequence. After clicking "Next", the properly translated sequence is given, as shown in Fig. 2A, The user then double-clicks the amino acid he wishes to mutate and selects from a drop-down list which residue it should be changed to. If the user wishes to mutate more than one amino acid, he can simply repeat this step. Each mutation is highlighted in red. In the next window, the user can select which restriction sites the program should search for. Currently, SiteFind has 131 restriction sites to choose from and uses them all by default, but the user is free to remove any of these or add new ones if so desired. Any restriction sites present in the wild-type sequence are removed from the search. The next window then displays a progress bar as it searches: in most cases, the search takes no more than a few seconds. Once finished, the user can click "Next" one last time, and the results are printed in a list. A list of potential restriction sites is given, For each site, the wild-type sequence displayed, with the necessary mutant sequence displayed just under it. Any differences between the two sequences are indicated. Below the mutant sequence, the location of the introduced restriction site is clearly marked. If there are multiple locations in the sequence where a given restriction site can be introduced, only the location closest to the desired point mutation is displayed. (See Fig. 2B). The user can then use this information to design the appropriate primers for performing site-directed mutagenesis.

### Examples of its use

We used this tool routinely in our laboratory. For example, Krüppel-like factor 4 (KLF4) is a transcription factor implicated in colon cancer. Previous studies on KLF4 have shown that a single point mutation, R390S, can abolish its ability to enter the nucleus, where it is normally exclusively located [13,14]. In order to make such a construct, we entered the wild-type DNA sequence corresponding to amino acids 385–393 into SiteFind and then specified the desired mutation R390S. Using the default settings, SiteFind found 10 restriction sites that we could use as a marker. We chose *BglII *since no *BglII *site was present in our original construct, and it required the mutation of only three nucleotides. Using this information, we were then able to design the proper primers for site-directed mutagenesis.

**Figure 3:**
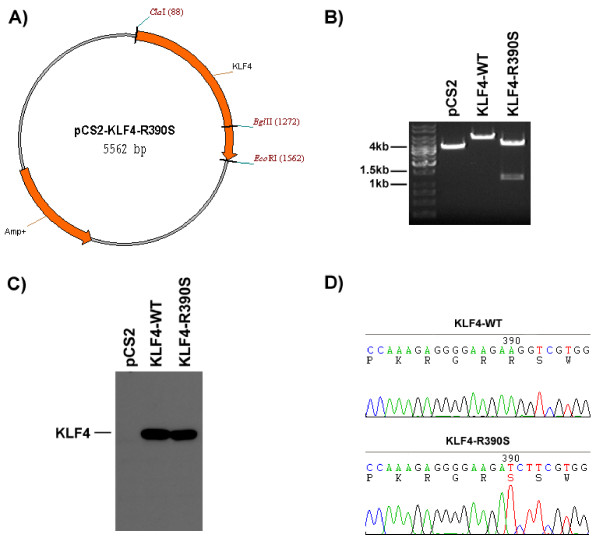
**KLF4 R390S mutant has a novel BglII restriction site**. a) pCS2-KLF4-R390S construct diagram. b) ClaI / BglII Restriction digest of both wild-type and successfully mutated plasmid DNA. c) α-Flag Western blot showing expression of mutant construct in 293T cells. d) Sequencing result of the mutation, mutated residue is highlighted in red.

After transformation of competent bacteria with the PCR product, we plated the cells on ampicillin-containing agar overnight. We then picked several colonies and isolated their plasmid DNA. The plasmid DNA was then digested with *ClaI*, which is present in the vector backbone, and *BglII*. Since *BglII *is neither present within the vector backbone nor the wild-type KLF4 sequence, *BglII *should only cut successfully mutated plasmid DNA, yielding a 1244 bp fragment (See Fig. 3A). As shown in Fig. 3B, wild-type plasmid DNA yields only one fragment, whereas successfully mutated DNA yields a second, 1.2 kb fragment.

To confirm that our mutant construct is expressed, we transfected 293T cells, lysed the cells 48-hours post-transfection, and performed an α-Flag Western blot with the lysate. Fig. 3C demonstrates that both the wild-type and mutant constructs express a protein of identical size, whereas transfection with an empty vector yields no Flag-tagged protein whatsoever. This is expected since a point mutation should have no detectable effect on the molecular weight. Finally we verified the mutant construct by sequencing (See. Fig. 3D).

**Figure 4:**
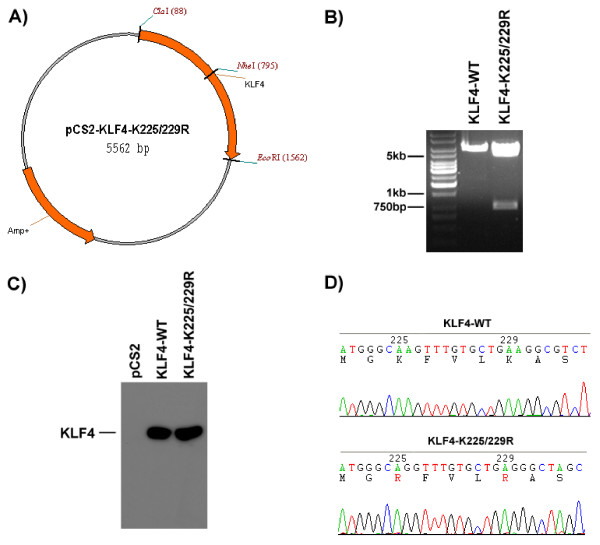
**KLF4 K225/229R mutant has a novel NheI restriction site**. e) pCS2-KLF4-K225/229R construct diagram. f) NheI / EcoRI Restriction digest of both wild-type and successfully mutated plasmid DNA. g) α-Flag Western blot showing expression of mutant construct in 293T cells. h) Sequencing result of the mutation, mutated residues are highlighted in red.

To demonstrate that SiteFind can be used to design multiple point mutation, we produced a double point mutation of KLF4, mutating two successive lysines (K225/K229) to arginine. Using SiteFind, we decided to introduce an *NheI *site just 3' to the second point mutation. After PCR and plasmid purification, we digested the mutant construct with *NheI *and *EcoRI*. *NheI *should only cut the mutant construct, producing a 767 bp fragment (See Fig. 4A). As expected *NheI *cuts the mutant construct to produce a second fragment of approximately 750 bp, whereas the wild-type plasmid yields only one fragment (See Fig. 4B). We confirmed expression of this construct in 293T cells, and as expected, both wild-type and K225/229R mutant KLF4 produce bands of identical size (See Fig. 4C). Finally, we verified our construct by sequencing (See Fig. 4D).

## Discussion

There are several programs available for designing primers for site-directed mutagenesis. Most of these programs are used to calculate the annealing temperature and to predict secondary structures. They cannot be used to design a restriction site. SiteFind is designed specifically for this.

In an easy-to-use, graphical interface, the user is prompted to enter the desired template nucleotide sequence. Then, the translated amino acid sequence is given and the user is able to select which amino acids to mutate. After that, the user can specify which restriction sites to search for, and even add additional sites if so desired. Finally, after a few seconds, a list of potential restriction sites is given. For each site, only the location closest to the desired point mutation and involving the fewest number of mutations is given. This substantially reduces the amount of information the user has to process prior to selecting the optimal sequence for site-directed mutagenesis, saving both time and money. Furthermore, SiteFind can be used for any type of mutagenesis and places no limits on the number of point mutations in the mutant sequence.

As the sequence length increases, when simply generating every possible nucleotide sequence for a given amino acid sequence and then searching for the presence of a restriction site, the time required for the search increases exponentially. If done in this manner, searches of longer than 15 bp quickly become infeasible. Our "moving window" algorithm is a novel way to drastically reduce the time required for a search, and yet does so without missing any potential sites. Because SiteFind implements this algorithm, it can process sequences up to 400 bp.

Shankarappa et. al. have published a computer program called SILMUT [9]. SILMUT is a simple command-line program that can search a short amino acid sequence for the 30 most common, 6 bp restriction sites. It does this by translating each restriction site in all three frames and compares every possible translation with the user-specified amino acid sequence. During preparation of this manuscript, we discovered another web-based program that performs a function similar to SiteFind, called the Primer Generator [15]. However, the Primer Generator requires the user to manually type in both the wild-type sequence and desired mutant amino acid sequence and to manually pick from hundreds of output sequences. Furthermore, it is not suitable for nucleotide sequences longer than 15 bp.

In contrast, SiteFind, automatically translates the input nucleotide sequence and allows the user to graphically select which residues to mutate. Furthermore, our window algorithm enables SiteFind to quickly and efficiently work with sequences of any length. For each restriction site, if multiple locations are found, SiteFind only gives the location closest to the desired point mutation: this means much less information for the user to parse in order to choose the best restriction site and sequence. Although not specifically designed for it, SiteFind could be used to make translational fusions between two different coding sequences. The user can specify that SiteFind give every location found for each restriction enzyme, and then run a search on a portion of both sequences. Then, through manual comparison, the user could select a restriction site found within both sequences and design the appropriate primers for introducing the necessary mutations.

## Conclusion

SiteFind is a useful tool for performing site-directed mutagenesis, enabling the user to introduce a novel restriction site into the mutated nucleotide sequence for use as a marker of successful mutation. The "moving window" is a novel algorithm that enables SiteFind to work efficiently with sequences up to 400 bp. In order to demonstrate its utility, we introduced a point mutation, R390S, into the wild-type sequence of KLF4 while simultaneously introducing a novel *BglII *restriction site. This mutant DNA could be cut by *BglII*, as expected, and expressed a full-length protein in 293T cells. For a double point-mutation, K225/229R, we introduced a novel *NheI *restriction site. This mutant DNA could be cut by *NheI*, as expected, and expressed a full-length protein in 293T cells.

## Materials and methods

### Materials

pCS2-Flag-KLF4 was sub-cloned from pMT3-KLF4, kindly provided by Dr. Vincent Yang, and verified by sequencing (MCLab, San Francisco, CA). All restriction enzymes and ligase were obtained from New England BioLabs (Ipswich, MA). Anti-Flag monoclonal antibody (m2) was purchased from Sigma (St. Louis, MO).

### Mutant preparation

SiteFind identified a potential *BglII *sequence overlapping with our desired R390S mutation of the KLF4 wild-type sequence [GenBank: BC010301]. Using the primer design guidelines included in the QuikChange II Site-Directed Mutagenesis Kit (Stratagene, La Jolla, CA), we chose forward primer 5'-CCAAAGAGGGGAAGATCTTCGTGGCCCCGG and reverse primer 5'- CCGGGGCCACGAAGATCTTCCCCTCTTTGG (*BglII *restriction site underlined). All primers were synthesized by Sigma-Genosys. PCR was performed using the *Pfu *Turbo DNA Polymerase (Stratagene, La Jolla, CA) according to manufacturer's instructions. The PCR product was then digested with *DpnI *to remove template DNA, followed by transformation of XL-10 competent bacteria. Bacteria were then plated on ampicllin-containing Luria-Bertani (LB) agar overnight at 37°C. Individual colonies were then grown in LB/Ampicillin medium for 12 hours at 37°C, and plasmid DNA was extracted using the Qiaprep Miniprep Kit (Qiagen, Valencia, CA). Purified DNA was then digested with *BglII *and *ClaI *and then run on an 0.8% agarose gel for 30 min at 120 V. Successful mutants, as determined by the presence of a second, 1244 bp band were grown in 100 mL LB/Ampicillin overnight and plasmid DNA extracted using the Qiagen Midiprep Kit (Qiagen, Valencia, CA). Purified DNA was then verified by sequencing. For our second mutant construct, K225/229R, SiteFind identified a potential *NheI *sequence. For this mutation, we chose forward primer 5'- CTGATGGGCAGGTTTGTGCTGAGGGCTAGCCTGACCACCCCTGGC and reverse primer 5'-GCCAGGGGTGGTCAGGCTAGCCCTCAGCACAAACCTGCCCATCAG (*NheI *restriction site underlined). We used similar methods to produce this construct, except that successful mutants were identified by restriction digest with *NheI *and *EcoRI *instead, which yields a 767 bp band.

### Cell culture and western blot

HeLa and 293T cells were grown in DMEM media supplemented with 10% FBS and 1% penicillin/streptomycin, and split as needed. For Western blot, 293T cells were plated on a 12-well plate and transfected with 1ug of either pCS2 empty vector, pCS2-Flag-KLF4, pCS2-Flag-KLF4-R390S, or pCS2-Flag-KLF4-K225/229R using the calcium phosphate method. After 6 hours, the media was replaced and the cells allowed to grow for another 36 hours. Cells were lysed in standard RIPA buffer with 1% Triton X-100 and protease inhibitor cocktail. Lysate was boiled in SDS sample buffer and run on a 10% polyacrylamide gel at 180 V for 45 min, and transferred to an Immobilon membrane (Millipore, Billerica, MA) at 30 V overnight. After blocking in TBS-T with 5% milk for 1 hr, membrane was incubated with α-Flag primary antibody (1:1000) for 1 hr, washed, and incubated with α-mouse secondary antibody (1:10,000). Membrane was then visualized using ECL buffer and exposed to X-ray film.

## Availability and requirements

Project name: SiteFind

Project home page: 

Operating system: Platform independent (any system with Java installed)

Programming language: Java

Other requirements: SiteFind is freely available to both academic and commercial users as a webpage-embedded Java applet.

Source code: Available at 

## List of abbreviations used

**bp: **base pair

**DNA: **Deoxyribonucleic acid

**HTML: **Hypertext markup language

**kb: **One thousand nucleotide bases

**PCR: **Polymerase chain reaction

## Authors' contributions

PME wrote both versions of SiteFind and was responsible for drafting this manuscript. In addition, PME performed all the experiments, including all PCR, restriction digests, Western blots, and immunostaining. CL originally suggested the idea and supervised the project.
